# Examining the spatial congruence between data obtained with a novel activity location questionnaire, continuous GPS tracking, and prompted recall surveys

**DOI:** 10.1186/1476-072X-12-40

**Published:** 2013-09-11

**Authors:** Martine Shareck, Yan Kestens, Lise Gauvin

**Affiliations:** 1 Département de médecine sociale et préventive, Université de Montréal, 7101 Avenue du Parc, H3N 1X7 Montréal, QC, Canada; 2 Institut de Recherche en Santé Publique de l’Université de Montréal, Montréal, Canada; 3 Centre de recherche du Centre Hospitalier de l’Université de Montréal, 3850 St-Urbain, H2W 1T7 Montréal, QC, Canada; 4 Centre de recherche Léa-Roback sur les inégalités sociales de santé de Montréal, Montréal, Canada

**Keywords:** Activity location questionnaire, Activity space, GPS devices, Mobility, Prompted-recall survey, Spatial behaviour

## Abstract

**Background:**

Place and health researchers are increasingly interested in integrating individuals’ mobility and the experience they have with multiple settings in their studies. In practice, however, few tools exist which allow for rapid and accurate gathering of detailed information on the geographic location of places where people regularly undertake activities. We describe the development and validation of a new activity location questionnaire which can be useful in accounting for multiple environmental influences in large population health investigations.

**Methods:**

To develop the questionnaire, we relied on a literature review of similar data collection tools and on results of a pilot study wherein we explored content validity, test-retest reliability, and face validity. To estimate convergent validity, we used data from a study of users of a public bicycle share program conducted in Montreal, Canada in 2011. We examined the spatial congruence between questionnaire data and data from three other sources: 1) one-week GPS tracks; 2) activity locations extracted from the GPS tracks; and 3) a prompted recall survey of locations visited during the day. Proximity and convex hull measures were used to compare questionnaire-derived data and GPS and prompted recall survey data.

**Results:**

In the sample, 75% of questionnaire-reported activity locations were located within 400 meters of an activity location recorded on the GPS track or through the prompted recall survey. Results from convex hull analyses suggested questionnaire activity locations were more concentrated in space than GPS or prompted-recall locations.

**Conclusions:**

The new questionnaire has high convergent validity and can be used to accurately collect data on regular activity spaces in terms of locations regularly visited. The methods, measures, and findings presented provide new material to further study mobility in place and health research.

## Background

A shift towards integrating mobility in place and health research is occurring. That is, researchers are increasingly interested in understanding people’s spatial behaviour and their daily activity settings when studying the influence of environmental resources on health. This mobility shift extends existing research which often focuses solely on residential neighbourhoods. It stems from evidence indicating that people visit a diversity of places in their daily lives and that each of these locations may influence their health in unique ways [1,2].

Given that people are mobile, exposures in a variety of places in addition to residential neighbourhoods should be assessed when accounting for environmental influences on health. Environmental conditions, resources, and opportunities available in residential areas differ considerably from those measured in people’s other activity settings [3-6]. For example, Kestens *et al*. (2010) found that the average fast food outlet density in daily activity locations was twice that measured in residential neighbourhoods [4]. Characteristics of activity settings have also been shown to modify or confound the association between residential neighbourhoods and health [7]. In a study by Inagami *et al*. (2007), adjusting for non-residential deprivation reinforced the inverse relationship between residential deprivation and self-rated health [7]. Since contextual measures and research findings may be sensitive to different spatial delineations of context [8,9], integrating mobility and people’s regular activity locations, also known as their activity space [10], in place and health research merits further exploration.

Towards this end, several data collection tools are available such as travel or activity diaries, travel surveys, global positioning systems (GPS), and activity location questionnaires. Travel diaries require participants to register detailed information on all trips (location of origins and destinations, start and end times, purpose, etc.) for a given period of time in a diary. Although the information gathered and the timeframe covered (which has ranged from two days [1] to multiple weeks [11]) can be adapted to one’s research question, travel diaries are time consuming and impose a heavy burden on participants. As well, short observation periods preclude collection of routinely but less frequently visited locations. Data collected using diaries may also deviate systematically from actual behaviour since respondents tend to underreport small trips and trips that do not start or end at home [12].

Stemming from the field of transportation research, travel surveys have also been used in place and health research [4,5]. They consist of Computer- or Web- Assisted Telephone Interviews to recall trips made by an individual over a given period, usually 24 hours, preceding the interview. Contrary to travel diaries which are generally used in small samples, travel surveys can be used in very large samples. However, they can exclude important activity locations which were not visited the day preceding the interview, and thus only partially represent one’s regular activity space [4,5,13].

An alternative to travel diaries and surveys resides in passive data collection tools such as GPS incorporated into cellular phones or sensors [12,14-18]. GPS devices have the advantage of providing objective information on travel routes and activity locations. However, the limited time frame they normally cover (one to 10 days) and other issues such as compliance, limited battery performance, or losing the signal indoors may also preclude the identification of regular activity locations. Similarly, the amount of data collected can become overwhelming and data processing requires a high skill level even though novel GPS processing toolkits are being developed and disseminated [19].

Finally, various types of questionnaires have been used to collect information on the geographical location of people’s regular activity places. Going back as far as the 1950s [20], activity location questionnaires have been used in public health research [7,21,22]. Activity location questionnaires do not necessarily refer to a reference period but rather use specific activities such as work, studies, or shopping as the starting point from which to derive geographical information on regular activity patterns [7,21]. Alternative forms of questionnaires may require participants to list and describe all the places where they spent time in a given time frame [23] or to report whether they usually undertake specified activities ‘mainly inside’ , ‘partly inside’ , or ‘mainly outside’ their residential neighbourhood [22,24]. Activity location questionnaires are useful in providing a rapid assessment of places where people spend time. However, when not directly supported by interactive mapping tools, locational data such as addresses and cross streets may be difficult to transform into precise and valid geographic coordinates [25].

Thus, despite their relevance and increasing use in research on activity spaces and health, the psychometric properties of activity location questionnaires have not been examined. Poor validity in activity location reporting may lead to invalid assignment of environmental exposures based on these locations and subsequently undermine the validity of their associations with health outcomes [26]. The spatial congruence between people’s reported regular activity locations and the places where they actually undertake activities thus warrants further investigation.

## Objective

In this paper, we describe the development and validation of a new activity location questionnaire which allows for the collection of detailed information on regular activity locations. The paper is divided into two parts. First, we outline the steps followed to develop the activity location questionnaire including estimating its test-retest reliability in a pilot study. Second, we estimate convergent validity of the new self-report measure based on data from one-week GPS tracks, GPS-derived activity locations, and a prompted recall survey of visited locations.

## 1. Developing the questionnaire

### Structure and content of the questionnaire

To obtain information on regularly visited locations, the questionnaire was structured around ‘where’ participants conducted a series of pre-identified habitual activities. The routine aspect of activities was central to the design since the aim was to assess the places where people *regularly spent time* rather than to cover a broad range of places people visited *sporadically*. Authors MS and YK identified activity types based on a local travel survey [27], time-use studies [28,29], and similar questionnaires [7,21,22,30].

Travel diaries spanning long periods of time show a high level of regularity in the locations where people undertake daily life activities. In the German MobiDrive study for example, ten locations linked to nine general activity types accounted for more than 80% of activities performed by participants during the six-week data collection period [11,31]. MS and YK thus compiled a list of nine broad activity types which would cover people’s regular activity locations: (1) studying; (2) working; (3) grocery shopping; (4) physical activity or sports; (5) leisure activities; (6) spending the night or weekend; (7) dropping off/picking up someone; (8) meeting friends or relatives; and (9) other activities. For each activity type they engaged in, participants were asked to provide details about the location where the activity took place (e.g., name of place, address, closest intersection or landmark, neighbourhood, city) to allow for transforming information into geographic coordinates, i.e. geocoding. Respondents were not asked to refer to a specific time period, such as the past week or month, to report their regular activities.

### Pre-testing the questionnaire

The questionnaire was pre-tested first by evaluating its content validity through an independent panel of six experts in public health, geography, and sociology. Experts rated the relevance and clarity of each question on a three-point scale and provided open feedback on the overall questionnaire. Data were collected using a standardized grid. Mean relevance and mean clarity scores were computed and comments were synthesized.

The questionnaire was then pilot-tested for test-retest reliability to verify if participants’ responses were stable over time [32]. Thirty-one adults (51% women, 18 – 25 years-old) were recruited from the research team’s network as well as through ads posted in public places. Respondents completed the questionnaire twice at a two week interval and took part in a semi-structured interview following questionnaire completion at time 2.

For each activity type, information provided on the location where the activity took place (name, address, intersection, etc.) was compared between times 1 and 2. Test-retest agreement was defined as having reported information at times 1 and 2 which led to identification of the same location. Participants were from diverse socio-economic backgrounds and areas of the city. They reported conducting a total of 104 activities. There was test-retest agreement for 86.5% of locations associated with these activities. Higher agreement was found for study and work locations (94.7% and 100% agreement respectively) whereas agreement ranged between 73.3% and 87.9% for other activities and for grocery stores) (data not shown).

### Finalizing the questionnaire

Following assessment of content validity and test-retest reliability, final modifications were made to the questionnaire. To improve the flow between questions we included filter questions which consisted of first asking respondents if they conducted a given activity before being asked to report the location. In addition, after a detailed revision of experts’ comments as well as participants’ responses and interview data, only six of the initial nine activity types were included in the final questionnaire: studying, working, grocery shopping, physical activity or sports, leisure activities, and other activities. These activities were deemed most relevant by experts and participants to adequately encompass regular activity types in a large and diverse adult population. For example, none of the participants in the test-retest reliability study provided the location where they “dropped off/picked up someone”. This activity type was therefore discarded. Similarly, it was deemed more efficient to shorten the questionnaire by removing “spending the night/weekend” and “meeting friends or relatives” from the specific activity types and allow these activity types to be included in the “other activities” category. Finally, because certain activities may regularly take place in more than one location, as became obvious upon reviewing participants’ responses and interview data, we allowed space for providing two locations for work, grocery shopping, and other activities. In the final version of the questionnaire which is available online (http://www.spherelab.org), respondents can thus report on their residential location as well as a total of nine locations where six activity types are performed.

## 2. Examining convergent validity

In a second step, we assessed the convergent validity of the final version of the questionnaire using data from a study of users of a public bicycle share program (BIXI) in Montreal, Canada. We compared the geographical location of activities reported in the questionnaire with data from three related data sets: 1) GPS tracks; 2) GPS-derived activity locations; and 3) online prompted recalls of locations visited during the day. We examined the spatial congruence between questionnaire-reported activity locations and those included in each data set using distance and convex hull (i.e. the smallest convex polygon encompassing all activity locations) size and overlap measures. These are described below.

### Methods

#### ***Data collection***

Thirty-nine volunteers were recruited as part of a wider cross sectional study of BIXI users (see [33] for details). Participants were asked to carry a cell-phone with an integrated GPS receiver for a period of eight days. A smaller sample size was preferred over a larger one since it allowed us to perform intensive ambulatory monitoring to better establish the feasibility and validity of our methodology. The GPS units were programmed to collect latitude, longitude, and local time every second. Tracklog data were regularly and automatically uploaded through the cell-phone network to a central server.

At the end of each day, participants were instructed to complete a prompted-recall survey. They would log to an online application called Mobility Web Mapping (MWM) where they could visualize their own GPS track for the day. Visualization of their GPS track prompted participants to recall the locations they had visited during the day. They were asked to identify their visited locations by positioning map markers and providing complementary information on trips such as arrival/departure times and transportation modes. This procedure provided a prompted-recall database of locations that were reportedly visited.

Prior to data capture, participants were offered a 30-minute training session on GPS-enabled cell phones and on the online prompted-recall application. At the beginning of the training session, participants self-administered a paper copy of the activity location questionnaire. Ethics approval was obtained from the Human Research Ethics committee of Centre Hospitalier de l’Université de Montréal.

#### ***Activity space data sources***

A total of four spatial datasets were available for comparison: (i) activity locations from the questionnaire; (ii) GPS tracks; (iii) GPS-derived activity locations; and (iv) activity locations reported through the prompted-recall survey.

*Activity location questionnaire (‘questionnaire locations’)*: Responses from the activity location questionnaire were cleaned and geocoded. Geocoding accuracy is maximized for exact street addresses so these were sought for reported activity locations using the Google© and GoogleMaps© search engines. When too few details were available to identify the exact address, the closest intersection or the place name were used for geocoding (respectively 3.7% and 2.2% of all locations reported). Latitude and longitude coordinates were obtained using a free, online geocoder [34].

*GPS tracks (‘GPS tracks’):* Raw GPS tracks were cleaned to remove data points with high dilution of precision (DOP) values, i.e. poor precision due to the low number and poor configuration of satellites. Points with Horizontal DOP > 8, Vertical DOP > 15 or Positional DOP > 13 were removed. GPS tracks are rarely continuous because of signal loss due to non visibility of satellites particularly inside homes or buildings. Missing GPS data points were thus imputed through linear interpolation between two points for any gaps of up to 60 minutes. For gaps of over 60 minutes, linear interpolation was performed if two consecutive data points were less than 100 meters apart. These cleaned, interpolated GPS tracks provided continuous (1 second epoch) monitoring of mobility.

*GPS-derived activity locations (‘GPS activity locations’)*: An activity location extraction algorithm [19] was applied to the interpolated GPS tracks providing locations and timetables (i.e. history of visits) for all activity locations. Stops of 5 minutes or more were retained as significant activity locations. Shorter stops, although detected, were discarded for the present analysis.

*Prompted-recall survey (‘prompted recall locations’)*: Online self-reports of locations visited collected through the MWM prompted-recall survey were obtained during the GPS tracking period and automatically geocoded.

#### ***Data analysis***

Questionnaire locations, GPS tracks, GPS activity locations, and prompted recall locations were analyzed in ArcGIS© v.10. We performed two types of analyses to compare questionnaire locations with these three datasets (two-by-two comparisons): proximity analyses and analyses using convex hulls.

*Proximity analyses:* For each participant, the Euclidian distance separating questionnaire locations from their closest neighbour in each of the other data sets was calculated in meters (m.).

*Convex hull analyses*: Questionnaire, GPS, and prompted recall data were also compared using a geometrical measure of activity space - the convex hull - an indicator of the spatial extent and dispersion characteristics of respondents’ activity patterns [35]. Convex hulls are defined as the smallest convex polygon encompassing all activity locations and were created for each participant and each dataset. The size of convex hulls was computed in meters squared (m^2^) and compared. The spatial overlap between the questionnaire-derived convex hull and convex hulls obtained from the GPS tracks, GPS activity locations, and prompted recall locations were also computed and expressed as a percentage of (1) the questionnaire-derived convex hull and (2) GPS tracks, GPS activity locations, or prompted recall locations convex hulls.

Median distances, convex hull sizes, and percentage overlaps were calculated, along with 25^th^ and 75^th^ percentiles given that variables were not normally distributed. Statistical analyses were performed with SPSS© v.20.

### Results

Thirty-nine participants agreed to participate in the study and completed the questionnaire. At the end of the data collection period, 32 participants had GPS data and 35 had responded to the prompted recall survey. Analyses based on GPS data were limited to participants who had between four and 10 days of GPS data (n = 23) whereas analyses based on the prompted recall survey included participants who had reported visiting at least four places during the data collection period (n = 31). Inclusion criteria were based on respect of study guidelines, evidence of compliance with data collection tools, and the necessity to have at least three activity locations for convex hull analyses to be performed.

Table 1 shows descriptive statistics for two subsamples, i.e. those who completed the questionnaire and were included in the GPS analyses (n = 23) or provided prompted recall data (n = 31). In each subsample, participants provided details for an average of 6.4 (SD: 1.3) and 6.1 (SD: 1.6) activity locations in the questionnaire. Participants included in the GPS analyses provided data for a mean of 7.7 days (SD: 1.3) which translated in the detection of a mean of 12.7 activity locations (SD: 10.7) whereas those included in the prompted recall survey analyses had recorded, on average, 12.9 activity locations (SD: 7.4).

**Table 1:** Descriptive information on adults who provided questionnaire and GPS (n = 23) or prompted recall information (n = 31) on activity locations in Montreal, Canada in 2011

	**Questionnaire + GPS data (n = 23)**	**Questionnaire + prompted recall locations (n = 31)**
**Socio-demographic characteristics**		
Sex (female),% (n)	43.5 (10)	38.7 (12)
Age, mean years (SD)	37 (12)	37 (11)
Education level, % (n)		
High school/trade school	4.3 (1)	3.2 (1)
College	21.7 (5)	19.4 (6)
Undergraduate	26.1 (6)	32.3 (10)
Graduate	47.8 (11)	45.2 (14)
Occupation, % (n)		
Student	21.7 (5)	19.4 (6)
Freelancer	13.0 (3)	9.7 (3)
Part-time employed	13.0 (3)	9.7 (3)
Full-time employed	52.2 (12)	61.3 (19)
Annual household income, % (n)		
< 20,000$	26.0 (6)	19.4 (6)
20,000, 50,000$	13.0 (3)	16.2 (5)
50,000, 75,000$	21.7 (5)	19.4 (6)
75,000, 99,000$	26.1 (6)	25.8 (8)
> 100,000$	8.6 (2)	12.9 (4)
No answer	4.3 (1)	6.5 (2)
**Mobility potential**		
Has driver’s license (yes), % (n)	73.9 (17)	80.6 (25)
Has access to a car (yes), % (n)	21.7 (5)	25.8 (8)
Has car-sharing membership (yes), % (n)	17.4 (4)	19.4 (6)
**Activity-related characteristics**		
Questionnaire locations		
Min, max	4, 8	3, 9
Mean (SD)	6.4 (1.3)	6.1 (1.6)
Days with GPS data		
Min, max	5, 9	---
Mean (SD)	7.7 (1.3)	---
GPS activity locations		
Min, max	3, 49	---
Mean (SD)	12.7 (10.7)	---
Prompted recall locations		
Min, max	---	4, 35
Mean (SD)	---	12.9 (7.4)

#### ***Proximity analyses: questionnaire vs. GPS tracks***

Medians and interquartile ranges (IQR) for the distance separating questionnaire locations from the closest point on the GPS track are included in Table 2. When considering all activity purposes combined, fifty percent of questionnaire locations were within 5 m (IQR = 1 m, 24 m) of a point on the GPS track. Stratifying by activity purpose, median distances ranged from 0.7 m (IQR = 0.3 m, 10 m) for secondary work locations to 16 m (IQR = 6 m, 37 m) for primary grocery shopping stores.

**Table 2:** Distance separating questionnaire activity locations from GPS tracks and GPS activity locations (n = 23) and prompted recall locations (n = 31) collected in Montreal, Canada in 2011

	**Distance in meters separating a questionnaire location from …**					
	**… closest point on GPS track (n = 23)**		**… closest GPS activity location (n = 23)**		**… closest prompted recall location (n = 31)**	
**Questionnaire activity purpose**	**n**^**a**^	**Median (IQR**^**b**^**)**	**n**	**Median (IQR)**	**n**	**Median (IQR)**
All purposes	23	5 (1, 24)	23	90 (27, 382)	31	9 (1, 146)
Home	23	1 (0.4, 3)	23	22 (13, 45)	31	1 (1, 2)
Studies	8	5 (1, 24)	8	77 (39, 118)	12	111 (6, 291)
Work1	19	3 (1, 5)	19	65 (35, 101)	24	1 (0.09, 21)
Work2	5	0.7 (0.3, 10)	5	22 (9, 1,411)	8	2 (1, 669)
Grocery shopping1	22	16 (6, 37)	22	140 (58, 387)	31	19 (1, 197)
Grocery shopping2	20	13 (4, 65)	20	334 (125, 488)	23	110 (47, 371)
Sports	16	2 (1, 21)	16	202 (25, 573)	20	56 (1, 214)
Leisure	10	7 (1, 200)	10	169 (24, 778)	12	26 (2, 167)
Other1	15	14 (1, 347)	15	96 (21, 762)	19	19 (0.08, 398)
Other2	9	10 (1, 510)	9	320 (39, 1476)	11	300 (16, 1248)

^a^number of participants having reported a given activity.

^b^IQR: Interquartile range = 25th percentile, 75th percentile.

#### ***Proximity analyses: questionnaire vs. GPS activity locations***

Table 2 also shows results from proximity analyses comparing questionnaire locations to GPS activity locations. Fifty percent of all locations reported in the questionnaire were within 90 m (IQR = 27 m, 382 m) of a GPS activity location whereas median distances ranged from 22 m (IQR = 13 m, 36 m) for residential location to 334 m (IQR = 125 m, 488 m) for secondary grocery shopping location when stratifying by activity purpose.

#### ***Proximity analyses: questionnaire vs. prompted recall locations***

Results pertaining to the comparison of questionnaire-based and prompted recall locations are also shown in Table 2. Fifty percent of questionnaire locations were situated within 9 m (IQR = 1 m, 146 m) of a location reported in the prompted recall survey. When stratifying by activity purpose, the median distance separating a questionnaire activity location from its closest neighbour in the prompted recall dataset ranged from 1 m for home and primary work locations (IQR = 1 m, 2 m and 0.09 m, 21 m respectively) to 300 m (IQR = 16 m, 1248 m) for secondary other activity locations.

#### ***Convex hull size analyses***

Results pertaining to convex hull sizes are presented in Table 3. Questionnaire convex hulls were generally smaller than those derived from the other data sources. For half of participants, the questionnaire convex hull was less than 4.1% (IQR = 1.2%, 41.5%), 27.7% (IQR = 1.9%, 74.9%), and 10.8% (IQR = 3.4%, 61.1%) of the size of their activity space as defined by their GPS tracks, GPS activity locations, and prompted recall locations respectively.

**Table 3:** Comparison of the size of convex hulls (CH) derived from questionnaire and CH derived from GPS (n = 23) and prompted recall data (n = 31) collected in Montreal, Canada in 2011

	**CH Size (km**^**2**^**)**	**Ratio of CH sizes: questionnaire to comparison data source**
	**Median (IQR**^**a**^**)**	**Median (IQR)**
**Questionnaire and GPS (n = 23)**		
Questionnaire activity locations	2. 6 (1.0, 6.3)	N.A
GPS tracks	27.8 (11.7, 186.9)	4.1 (1.2, 41. 5)
GPS activity locations	5.9 (2.1, 58.7)	27.7 (1.9, 74.0)
**Questionnaire and prompted recall survey (n = 31)**		
Questionnaire activity locations	2.6 (1.0, 6.7)	N.A
Prompted recall activity locations	15.5 (6.0, 69.2)	10.8 (3.4, 61.1)

^a^IQR: Interquartile range = 25th percentile, 75th percentile.

#### Convex hull overlap analyses

**Figure 1:**
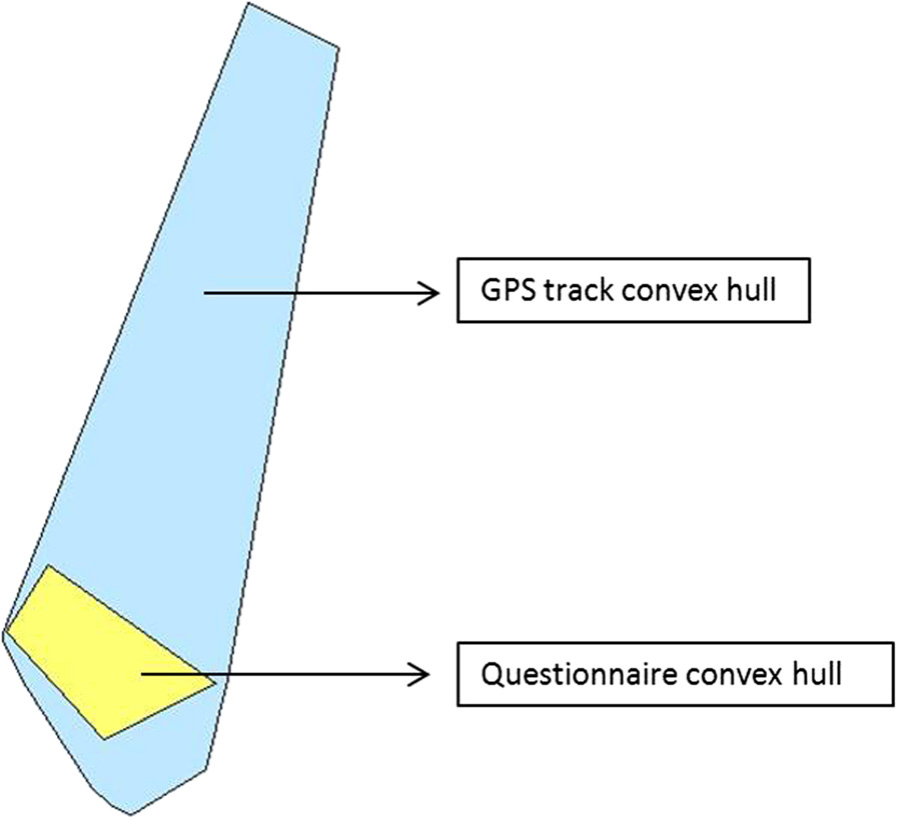
**Comparison of convex hull sizes and description of overlaps: questionnaire and GPS track convex hulls in data collected in Montreal, Canada in 2011.** The questionnaire convex hull (in yellow) and GPS track convex hull (in blue) overlap so that the overlapping area is 100% the size of the questionnaire convex hull, and 12% the size of the GPS track convex hull.

Table 4 presents results concerning convex hull overlap measures. Fifty percent of participants had their questionnaire convex hull almost completely or completely encompassed by the convex hull formed by their GPS tracks and prompted recall locations. To illustrate this finding, we provide an example in Figure 1 where the percentage overlap between questionnaire and GPS track convex hulls would be 100% of the questionnaire convex hull but only 12% of the GPS track convex hull.

**Table 4:** Spatial overlap between questionnaire-derived convex hulls (CH) and GPS (n = 23) and prompted recall convex hulls (n = 31) based on data collected in Montreal, Canada in 2011

	**Spatial overlap as % of questionnaire CH**	**Spatial overlap as % of comparison data source CH**
	**Median (IQR**^**a**^**)**	**Median (IQR)**
**Questionnaire and GPS (n = 23)**		
Questionnaire activity locations	N.A	N.A
GPS tracks	100 (94.7, 100)	4.0 (1.2, 25.6)
GPS activity locations	78.5 (37.1, 98.1)	11.9 (1.4, 49.8)
**Questionnaire and prompted recall survey (n = 31)**		
Questionnaire activity locations	N.A	N.A
Prompted recall activity locations	99.3 (89.1, 100)	10.7 (3.4, 57.7)

^a^IQR: Interquartile range = 25th percentile, 75th percentile.

Median overlaps expressed as the percentage of the questionnaire convex hull area were indeed 100% (IQR = 94.7%, 100%) and 99.3% (IQR = 89.1%, 100%) for GPS tracks and prompted recall convex hulls respectively (Table 4). The median overlap between convex hulls formed by GPS and questionnaire activity locations reached 78.5% (IQR = 37.1%, 98.1%) when expressed as a percentage of the area covered by questionnaire convex hulls. When expressed as a percentage of convex hulls derived from GPS tracks, GPS activity locations or from prompted recall locations, median overlap was much lower, ranging between 4.0% (IQR = 1.2%, 25.6%) and 11.9% (IQR = 1.4%, 49.8%).

## Discussion

In this paper, we described the development and validation of an activity location questionnaire which allows for the assessment of the places where people regularly conduct activities. Pilot testing the questionnaire for content validity, test-retest reliability, and face validity allowed for improvement of the questionnaire. Following this pre-test phase, six types of activities in which people regularly engage were included in the final version of the questionnaire.

To estimate the new tool’s convergent validity, we compared the geographical location of questionnaire-reported activities to (1) GPS tracks; (2) GPS activity locations; and (3) daily prompted recalls of visited locations using proximity and convex hull size and overlap measures. Although travel surveys have been tested against GPS data to estimate accuracy in terms of trip reporting [36], this study is, to our knowledge, the first to examine the spatial congruence between regular activity locations reported in a questionnaire and activity locations collected with alternative tools. This study allowed for examination of the spatial match (or mismatch) between regular activity locations reported in a questionnaire and people’s spatial behaviour as depicted by data from GPS devices and by a self-reported assessment of visited locations, i.e., prompted recalls.

Small distances separating regular activity locations reported in the questionnaire and locations recorded with the alternative tools suggested spatial congruence between data sources. Results from proximity analyses suggested that 75% of questionnaire-reported activity locations were within a distance of less than 400 meters from a GPS activity location or prompted recall location. More interestingly, analyses based on the GPS tracks suggested that participants had been active within even shorter distances from the regular activity locations they had reported in the questionnaire: a GPS point had been recorded within 24 m of 75% of questionnaire locations. These findings provide evidence supporting the idea that a self-administered questionnaire can be used to collect accurate data on regular activity locations since most questionnaire locations were within short distances from data points or activity locations collected with GPS devices and prompted recall surveys.

We found discrepancies between questionnaire data and the alternative data sources as seen by convex hull sizes and overlaps. Questionnaire-derived convex hulls were considerably smaller than convex hulls based on GPS tracks, GPS activity locations, and prompted recall locations. As well, more than half of participants had their questionnaire-derived activity space completely or almost completely encompassed within their GPS or prompted recall activity space. Thus, although the questionnaire-based activity space fell within the lived space as defined by GPS and prompted-recall data, it did not represent the full spatial extent of activities collected with these alternative tools. These results are not completely unexpected and can be explained by regular activity locations generally being more concentrated in space than the complete set of places people actually visit during a day or week, which is closer to what was measured with the GPS and prompted recall survey. In fact, regular activity locations tend to cluster spatially around few focal points such as the home or workplace even though people move around and travel longer distances from time to time [35].

The lack of proximity between certain questionnaire-reported activity locations and locations found in the comparison datasets and the mismatch in terms of convex hull sizes and overlaps could also be attributed to a number of factors which unfortunately could not be disentangled here. First, errors when geocoding questionnaire, GPS data and prompted recall locations may have occurred given the imprecisions inherent to geocoding tools: positional errors have been suggested to vary between 58 m and 96 m in urban areas [37]. These distances are small enough to assume that positional errors would not have significantly impacted results. There may also have been technical problems with the cell-phone integrated GPS devices resulting, for example, in lost data and undetected activity. The criteria we established for GPS data interpolation, which could have located individuals in places they never visited, as well as to define GPS-derived activity locations, may also have introduced error which could explain some of the larger distances found between questionnaire locations and GPS-derived locations.

Second, the regular activity locations reported in the questionnaire may simply not have been visited during the data collection period. This mismatch is understandable given that we compared data collection tools which are not meant to provide exactly the same spatial information. Seven days (minus or plus three days) may also be too short a period to encompass all the activities people conduct regularly. For example, someone might have reported regularly going to a vacation home on week-ends but simply did not go during the data collection period. Alternatively, since the term “regular activity” was not defined in the questionnaire, participants may have judged it rigidly and consequently underreported certain activities which, although conducted frequently were not considered regular or routine activities. In the future, to limit variability in interpreting the meaning of “regular”, it could be useful to provide a time frame which participants could refer to when reporting activities.

This study has limitations which should be acknowledged. First, study participants tended to be of working age, fairly affluent, and to live close to the city center. Results may therefore not be generalizable to other subgroups such as the elderly or suburban residents who have been found to be respectively less and more mobile than the study population [38]. The small sample size also hampered us from moving beyond descriptive statistics. Third, there might have been issues with low compliance with online prompted recalls which could have led to an underreporting of locations visited during the data collection period. Although compliance levels could not be estimated, we attempted to limit the impact of such underreporting on results by only including participants who had reported visiting at least four locations in the prompted recall survey. Fourth, it was beyond the scope of this paper to examine the correspondence between activities reported in the questionnaire and those recorded by GPS or in the prompted-recall survey in terms of their *purpose*. It is therefore not possible to determine if the exact questionnaire-reported activity locations were used for their stated purpose during the data collection period. Finally, we compared the questionnaire to three data sets which were not independent from one another: recalls were prompted by the visualization of the daily GPS track, and GPS activity locations were extracted from this same track. This should be kept in mind when interpreting results.

## Conclusion

This study contributes to positioning activity location questionnaires as valid alternatives or as complements to GPS and surveys in place and health research. It provides needed information regarding the psychometric properties of tools to collect data on people’s activity and spatial patterns. The activity location questionnaire presented here had high convergent validity, defined as the spatial congruence between questionnaire locations and activity locations collected with alternative tools, and could be used in larger studies. In addition, the methods and measures described are unique and novel and could be applied to other datasets to compare spatial information from various sources.

### Consent

Written informed consent was obtained from participants prior to data collection.

## Competing interests

The authors declare having no competing interests.

## Authors’ contributions

MS was responsible for developing the questionnaire, conducting data analysis, interpreting results, and writing the manuscript. MS, YK, and LG conceptualized and designed the study. YK and LG provided critical feedback on all versions of the manuscript. All authors have read and approve the final manuscript.
